# Management of Cardiovascular Implantable Electronic Device Infection Utilizing a Multidisciplinary Team: A Retrospective Cohort Study

**DOI:** 10.1093/ofid/ofaf148

**Published:** 2025-03-10

**Authors:** Evan Hall, Bennett Collis, Talal Alnabelsi, Chloe Cao, Meredith Johnson, John Gurley, Luke Strnad, Hassan Reda, Tessa London, Erinn Ogburn, Michael Sekela, Bobbi Jo Stoner, Sami El-Dalati

**Affiliations:** University of Kentucky College of Medicine, Lexington, KY, USA; University of Kentucky College of Medicine, Lexington, KY, USA; Division of Cardiology, Department of Internal Medicine, University of Kentucky Medical Center, Lexington, KY, USA; Department of Internal Medicine, HCA Healthcare/USF Morsani College of Medicine GME/HCA Florida Trinity, Tampa, FL, USA; Department of Surgery, University of Kentucky Medical Center, Lexington, KY, USA; Division of Cardiology, Department of Internal Medicine, University of Kentucky Medical Center, Lexington, KY, USA; Division of Infectious Diseases, Department of Internal Medicine, Oregon Health and Science University, Portland, OR, USA; Division of Cardiac Surgery, University of Kentucky Medical Center, Lexington, KY, USA; Division of Cardiac Surgery, University of Kentucky Medical Center, Lexington, KY, USA; Division of Cardiac Surgery, University of Kentucky Medical Center, Lexington, KY, USA; Division of Cardiac Surgery, University of Kentucky Medical Center, Lexington, KY, USA; University of Kentucky College of Pharmacy, Lexington, KY, USA; Division of Infectious Diseases, Department of Internal Medicine, University of Kentucky Medical Center, Lexington, KY, USA

**Keywords:** cardiac implantable electronic device extraction, cardiac implantable electronic device infections, lead management teams, endocarditis teams, multidisciplinary teams

## Abstract

**Background:**

Significant numbers of cardiac implantable electronic devices are placed in the United States, and infection can be a severe consequence of implantation. Despite guideline recommendations and data demonstrating that lead extraction improves mortality for patients with device infection, rates of lead removal are lower than guideline recommendations. We report the outcomes associated with management of suspected cardiac implantable electronic device infections by a multidisciplinary team.

**Methods:**

Patient cases were identified from an institutional multidisciplinary endocarditis team registry in a single-center retrospective study. Demographic, treatment, and outcomes data were recorded by study investigators.

**Results:**

Between September 7, 2021, and February 1, 2024, 80 consecutive patients with suspected cardiac implantable electronic device infections were identified. Fifty-four (67.5%) patients, including 27/35 (77.1%) with definitive device infection/endocarditis and 30/30 (100%) with pocket infections, underwent device removal at a median of 3 days after admission to the authors’ institution. In-hospital and 90-day mortality was numerically lower in patients who underwent device removal compared with those who did not (13% vs 19.2%; *P* = .47 and 22.2% vs 38.5%; *P* = .13), and 1-year mortality was significantly lower for patients who underwent device removal (34% vs 70.1%; *P* = .01). Only 48.1% of patients had a device replaced during the study period.

**Conclusions:**

The use of a multidisciplinary team for management of suspected cardiac implantable electronic device infections was associated with high rates of expedient device extraction and relatively low rates of device reimplantation. One-year mortality was lower in patients who underwent device extraction.

Cardiovascular implantable electronic devices (CIEDs) are used routinely to manage the risk of life-threatening arrhythmias. CIEDs generally encompass transvenous pacemakers and implantable cardioverter defibrillators (ICDs) but have evolved over time and now include leadless pacemakers and subcutaneous ICDs. Over 400 000 CIEDs are implanted annually in the United States, with a lifetime incidence of infection ranging from 1.5% to 2.4% [1]. Infections of these devices (CIEDIs) account for many of the negative and potentially fatal consequences of implantation. Aside from subtle differences in the classification of diagnostic criteria, the American Heart Association, European Society of Cardiology, and European Heart Rhythm Association all agree that prompt removal is necessary once a CIEDI has been diagnosed, and in some cases of bloodstream infection with high-risk organisms, prompt removal is required even when the presence of a CIEDI is ambiguous [2–4]. While early (within 10 days of diagnosis) device extraction for CIEDI is associated with decreased mortality, over 85% of patients with indications do not undergo extraction [5, 6]. Optimal management is also often delayed, and low rates of timely extraction may be explained by common barriers such as delayed diagnosis and referral and misconceptions about the risks vs benefits of device removal [5–7]. Additionally, the limits in sensitivity of diagnostic imaging modalities such as transthoracic and transesophageal echocardiography and positron emission tomography computed tomography (PET-CT) may not allow for consistent identification of patients with CIEDI. Device removal is an intervention that requires specialized procedural skills not always present at small- or medium-sized hospitals. Lastly, the literature about whether device retention with antibiotic suppression can be successfully implemented in a subset of patients at high risk for extraction is limited and suggests a high morality associated with this approach [8]. Consequently, physicians are often left weighing the risks of device extraction against those of relapsed or progressive infection if a potentially infected CIED is retained. Currently, many infectious disease (ID) and cardiology providers have insufficient education to adequately assess both sets of risks [9]. Multidisciplinary teams have been demonstrated to improve clinical outcomes, including short-term mortality, for patients with infective endocarditis (IE) [10, 11]. However, there are few data on the impact of multidisciplinary teams for suspected CIEDIs. Here, we present our 2.5 years of experience managing suspected CIEDIs using a multidisciplinary team approach.

## METHODS

### Team Protocol

In September 2021, University of Kentucky Healthcare (UK) created a multidisciplinary endocarditis/CIEDI team (MDET) and cardiovascular infectious diseases consult service (CVIDCS). The CVIDCS is an interdisciplinary team housed in the division of infectious diseases and comprised of an attending physician, advanced practice provider, nurse navigator, pharmacist, and social worker. The CVIDCS receives a notification in the electronic medical record when a patient with a CIED is found to have a positive blood culture. The CVIDCS consults on all inpatients with suspected CIEDIs and follows them throughout the hospitalization and transition to outpatient. For the purposes of the CVIDCS, suspected CIEDI includes definitive pocket infection, possible CIED/infective endocarditis (CIED/IE), and definite CIED/IE. Patients with a CIED and *Staphylococcus aureus* bacteremia are also considered as suspected CIEDI. The CVIDCS coordinates the weekly MDET meetings and works with other specialties to arrange follow-up [8]. The MDET, in contrast, is composed of providers from infectious diseases, cardiac surgery, cardiology (including electrophysiology), addiction medicine, neurosurgery, neurology, physical medicine and rehabilitation, palliative care, and ethics. The group formally meets weekly to discuss all inpatients with suspected IE and CIEDIs and documents the meeting recommendations in the electronic medical record. Cases for the conference are identified primarily by the CVIDCS in collaboration with the cardiology and cardiac surgery services. All decisions regarding CIED lead management are made at the weekly MDET conference. This includes whether to perform device extraction, type and duration of antibiotic therapy, and the need for device reimplantation based on objective indications for CIED placement. Device removals are performed by an experienced cardiology extractionist who is also a member of MDET. Cardiac surgery support is provided for all removals that occur >1 year after device implantation.

### Patient Identification

Beginning in September 2021, a registry was created in the hospital's electronic medical record containing all patients presented at the weekly MDET conference. Institutional review board approval was obtained from the University of Kentucky to establish a database to retrospectively collect each patient's demographic, comorbidities, diagnostics, treatments, and outcomes data. Patient consent was not required.

**Figure 1.**
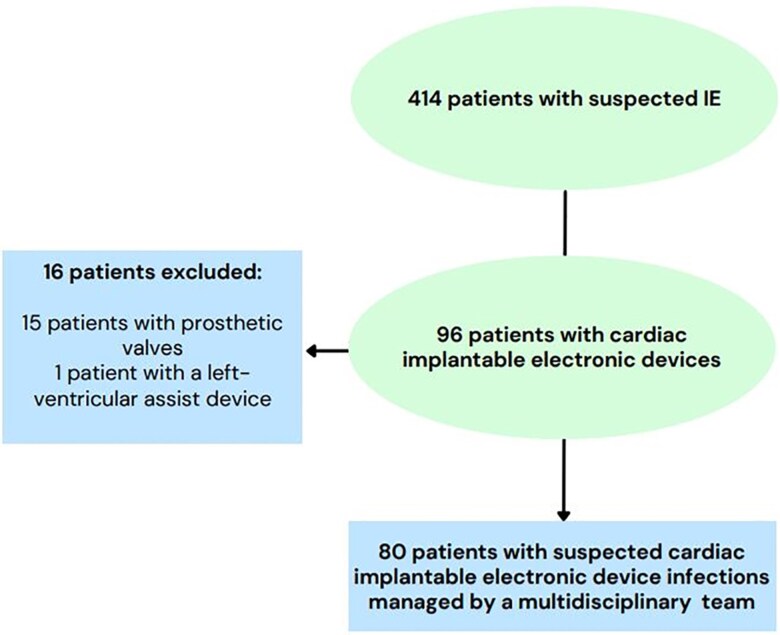
Study enrollment flowchart. Abbreviation: IE, infective endocarditis.

Patients were included in this study if they had a suspected or confirmed CIEDI as determined formally by the MDET. All patients with suspected CIEDIs were then reviewed by study investigators to classify their type of infection. Exclusion criteria included the presence of a prosthetic heart valve or left ventricular assist device (LVAD) (Figure 1). Patients with prosthetic valve endocarditis are typically managed with surgical redo valve replacement and concomitant device extraction. In patients who are not candidates for redo surgery, lead extraction is often deferred given concerns that it will not eradicate the infection entirely given that patients still have a retained prosthetic valve. LVADs are managed by the heart failure and transplant teams. Patients were separated into 2 groups based on whether their device was extracted or retained. The reporting of this study conformed to the Strengthening the Reporting of Observational Studies in Epidemiology statement [12].

### Definition of Terms

CIEDs were defined as transvenous, epicardial, and leadless pacemakers, as well as transvenous and subcutaneous ICDs. European Heart Rhythm Association diagnostic criteria were used to define the following categories: definite pocket infection, definite CIED/IE, possible CIED/IE, and rejected CIED/IE [3].

Persistent bacteremia was defined as blood cultures that were positive for ≥72 hours from the initial positive culture. Initial antibiotic course was defined as the duration of antimicrobials prescribed for primary treatment of the device infection and specifically excluded durations of antibiotics that were used for suppressive therapy. Oral suppressive antibiotics were defined as antimicrobials that were started after completion of an initial intravenous antibiotic course with the goal of preventing infection relapse rather than achieving cure of the index infection.

Acute renal failure was defined as patients who were initiated on renal replacement therapy during the index hospitalization.

Reinfections were defined as any patients who developed subsequent definite CIED/IE or definite pocket/generator infection after their index hospitalization and management by the MDET. Hospital readmissions were defined as readmissions occurring for any reason after discharge from the index hospitalization.

## RESULTS

### Study Population

Between September 7, 2021, and February 1, 2024, 80 patients with suspected CIED infection were identified. Demographic information was overall similar between the extraction and nonextraction groups (Table 1). Notable exceptions included a higher rate of diabetes (59.3% vs 30.8%; *P* = .02) and a higher proportion of White vs non-White patients in the extraction group (100% vs 88.5%; *P* = .01). More patients were bacteremic in the nonextraction cohort (92.3% vs 63%; *P* = .01). There were higher rates of *Candida* spp. isolated in the nonextraction cohort (11.5% vs 0%; *P* = .01) and higher rates of non-*Candida*/non-*Staphylococcal* spp. in the extraction cohort (42.5% vs 23.1%; *P* = .09). There were no significant differences between the groups with respect to when patients’ original CIEDs were placed. In both groups, suspected CIEDI was most common in patients whose devices were placed >5 years before the index hospitalization. There were more ICDs and cardiac contractility modulation (CCM) devices in those with device removal vs more permanent and leadless pacemakers in those with device retention (Table 1). Notably, there was no clear difference in collected markers of illness acuity in either group, although patients were frequently critically ill in both groups (40.7% extraction vs 34.6% retention; *P* = .60) and frequently transferred from outside hospitals (44.4% extraction vs 38.5% retention; *P* = .51).

**Table 1.** Demographic Information of Patients With Suspected Cardiac Implantable Electronic Device Infections Managed by a Multidisciplinary Team

Variable	Extraction Group (n = 54)	Nonextraction Group (n = 26)	*P* Value
Age, median (IQR), y	68 (17.5)	65 (26.8)	.65
Male, No. (%)	75.9 (41)	65.4 (17)	.33
Female, No. (%)	24.1 (13)	34.6 (9)	.33
White, No. (%)	100 (54)	88.5 (23)	.01
African American, No. (%)	0 (0)	11.5 (3)	.01
Injection drug use, No. (%)	1.9 (1)	11.5 (3)	.07
Prior endocarditis, No. (%)	7.4 (4)	7.7 (2)	.96
Hepatitis C, No. (%)	1.9 (1)	3.8 (1)	.61
Diabetes, No. (%)	59.3 (32)	30.8 (8)	.02
Dental disease, No. (%)	14.8 (8)	30.8 (8)	.10
Chronic dialysis, No. (%)	1.9 (1)	3.8 (1)	.61
Active malignancy, No. (%)	5.6 (3)	0 (0)	.22
Implantable cardioverter defibrillator, No. (%)	77.8 (42)	57.7 (15)	.06
Permanent pacemaker, No. (%)	22.2 (12)	34.6 (9)	.24
Leadless pacemaker, No. (%)	0 (0)	7.7 (2)	.04
Cardiac contractility modulation, No. (%)	3.7 (2)	0 (0)	.32
CIED placed <1 y ago, No. (%)	29.6 (16)	26.9 (7)	.80
CIED placed 1–5 y ago, No. (%)	24.1 (13)	34.6 (9)	.33
CIED placed >5 y ago, No. (%)	46.3 (25)	38.5 (10)	.51
Outside hospital transfer, No. (%)	44.4 (24)	38.5 (10)	.51
Length of outside hospital admission, median (IQR), d	3.5 (10.5)	4.5 (5.8)	.58
ICU stay, No. (%)	40.7 (22)	34.6 (9)	.60
Length of ICU stay, median (IQR), d	8 (9.5)	7 (5)	.19
Mechanical ventilation requirement, No. (%)	24.1 (13)	19.2 (5)	.63
Extracorporeal membrane oxygenation requirement, No. (%)	3.7 (2)	3.8 (1)	.98
Vasopressor requirement, No. (%)	16.7 (9)	23.1 (6)	.50
Acute renal failure requiring RRT, No. (%)	7.4 (4)	3.8 (1)	.54
Bacteremia, No. (%)	63.0 (34)	92.3 (24)	.01
Persistent bacteremia, No. (%)	22.2 (12)	30.8 (8)	.41
Pitt Bacteremia Score, median (IQR)	0 (2)	1 (2)	.18
*Staphylococcus epidermidis*, No. (%)	20.4 (11)	11.5 (3)	.33
Methicillin-resistant *Staphylococcus aureus*	24.1 (13)	23.1 (6)	.92
Methicillin-susceptible *Staphylococcus aureus*	13.0 (7)	30.8 (8)	.06
*Streptococcus* spp.	1.9 (1)	3.8 (1)	.61
*Enterococcus faecalis*	7.4 (4)	3.8 (1)	.54
*Pseudomonas* spp.	3.7 (2)	3.8 (1)	.98
*Klebsiella* spp.	1.9 (1)	3.8 (1)	.61
*Candida* spp.	0 (0)	11.5 (3)	.01
Culture negative	22.2 (12)	0 (0)	.01

Abbreviations: CIED, cardiac implantable electronic device; ICU, intensive care unit; IQR, interquartile range; RRT, renal replacement therapy.

### Diagnostic Evaluation

Diagnostic evaluation was similar between the 2 groups, with high rates of transthoracic echocardiography (TTE), lower rates of transesophageal echocardiography (TEE), and limited use of PET-CT (Table 2). A higher proportion of patients in the extraction group had a valvular vegetation, indicating the presence of concomitant native valve endocarditis (44.4% vs 15.4%; *P* = .01), which was mostly driven by tricuspid valve endocarditis. Over half of the extraction group (55.6%) had a definitive pocket infection compared with none of those in the device retention group. There was also a trend toward a higher proportion of patients with definitive CIED/IE (driven by the native valve endocarditis cases) in the extraction group and a higher proportion of possible CIED/IE in the retention group. Thirty-one percent (8) of patients with device retention met European Heart Rhythm Association (EHRA) criteria for definite CIED/IE, 34.6% (9) met criteria for possible CIED/IE, and 34.6% (9) were rejected by EHRA criteria.

**Table 2.** Imaging Modalities Utilized, Location of Cardiac Vegetations, and Categorization of Suspected Cardiac Implantable Electronic Device Infections Based on the European Heart Rhythm Association Diagnostic Criteria Patients With Suspected Cardiac Implantable Electronic Device Infections Managed by a Multidisciplinary Team

Variable	Extraction Group (n = 54)	Nonextraction Group (n = 26)	*P* Value
Transthoracic echocardiography, No. (%)	92.6 (50)	92.3 (24)	.96
Transesophageal echocardiography, No. (%)	50.0 (27)	46.2 (12)	.75
Cardiac positron emission tomography, No. (%)	0 (0)	3.8 (1)	.15
Lead vegetation, No. (%)	44.4 (24)	15.4 (4)	.01
Valvular vegetation, No. (%)	29.6 (16)	19.2 (5)	.32
Aortic valve vegetation, No. (%)	5.6 (3)	7.7 (2)	.72
Tricuspid valve vegetation, No. (%)	22.2 (12)	3.8 (1)	.04
Mitral valve vegetations, No. (%)	1.9 (1)	7.7 (2)	.21
Definite pocket/generator infection, No. (%)	55.6 (30)	0 (0)	<.0001
Definite CIED/IE, No. (%)	50.0 (27)	30.8 (8)	.11
Possible CIED/IE, No. (%)	13.0 (7)	34.6 (9)	.02
Rejected CIED/IE, No. (%)	37.0 (20)	34.6 (9)	.84

Abbreviations: CIED, cardiac implantable electronic device; CIED/IE, cardiac implantable electronic device/infective endocarditis.

### Management

In those who underwent device extraction, it was performed at a median of 3 days after admission to the authors’ institution and a median of 5 days after admission to any hospital (Table 3). Six patients (11.1%) had concomitant percutaneous mechanical aspiration of a lead or tricuspid valve vegetation at the time of CIED removal. Of the 20 patients in the extraction group with rejected CIED/IE, 18 met criteria for definite pocket infection. There were also 12 patients in the extraction group with possible or definite CIED/IE and concomitant definite pocket infection. Additional demographic and outcomes data for patients who underwent extraction for CIED/IE compared with those who underwent extraction for definite pocket infection are available in Supplementary Table 1.

**Table 3.** Device Management Strategies for Patients With Cardiac Implantable Electronic Device Infection Undergoing Extraction

Variable	Extraction Group (n = 54)
Days from admission to any hospital to extraction, median (IQR)	5 (8.8)
Days from admission to study hospital to extraction, median (IQR)	3 (5)
Percutaneous mechanical aspiration performed at the time of CIED extraction, No. (%)	11.1 (6)
Temporary pacing wire placement inpatient, No. (%)	5.6 (3)
New CIED placement after extraction, No. (%)	35.2 (19)
Days to new CIED placement after extraction, median (IQR)	3 (6)
Transvenous pacemaker placement inpatient, No. (%)	3.7 (2)
ICD placement inpatient, No. (%)	11.1 (6)
Leadless pacemaker placement inpatient, No. (%)	18.5 (10)
Life vest at discharge, No. (%)	14.8 (8)
ICD placement after index hospitalization, No. (%)	13.0 (7)
Days to new ICD placement postdischarge, median (IQR)	57 (36)

Abbreviations: CIED, cardiac implantable electronic device; ICD, implantable cardioverter defibrillator; IQR, interquartile range.

Sixteen (29.7%) patients’ CIEDs were placed within 1 year of the index hospitalization, and an additional 8 (14.8%) underwent lead or generator revisions within 6 months of the index hospitalization. A minority of patients (35.2%) underwent replacement of a new CIED during the index hospitalization at a median of 3 days after the extraction, and in over half of these, a leadless pacemaker was placed. Another minority (13.0%) had a new CIED replaced after the index hospitalization at a median of 57 days after extraction. Patients undergoing device removal received a median of 27 days of intravenous antibiotic therapy vs 17 days in the nonextraction group (Supplementary Table 2). A higher percentage of patients in the extraction group (37.0%) vs the nonextraction group (7.7%) transitioned to a median of 9.3 days of oral antibiotics to complete their primary treatment course. There was a higher percentage of patients in the nonextraction group placed on suppressive oral antimicrobials at the end of the primary treatment course (19.2% vs 3.7%).

### Clinical Outcomes

Five patients (9.3%) in the extraction group experienced a cardiac arrest, and all died during the index hospitalization compared with only 1 patient (3.8%) in the nonextraction group (Table 4). In contrast, more patients in the nonextraction group suffered a cerebrovascular accident (CVA; 15.4% vs 3.7%; *P* = .06). Of the 2 CVAs in the extraction group, 1 was attributed to concurrent mitral valve endocarditis, and no clear etiology was identified in the other case. Of the 4 CVAs in the nonextraction group, 3 were thought to be cardio-embolic, with atrial fibrillation as the primary risk factor in 2 cases and the presence of a left ventricular thrombus as the risk factor in the other. The remaining CVA was attributed to concurrent left-sided endocarditis. Although inpatient mortality was similar between the 2 groups, there was a trend toward higher 90-day mortality for patients whose devices were retained. The median length of stay was also similar between the extraction (11.0 days) and nonextraction groups (10.5 days). There was not a clear difference in reinfections or hospital readmissions between the 2 groups. One-year mortality data were not available for 6 patients overall: 4 from the extraction group and 2 from the nonextraction group. This was due to loss of follow-up and to some patients’ index admissions occurring within 12 months of the study. One-year mortality was 34% (out of 50 available patients) in the extraction group and 70.1% (out of 24 available patients) in the nonextraction cohort (*P* = .01).

**Table 4.** Clinical Outcomes for Patients With Suspected Cardiac Implantable Electronic Device Infections Managed by a Multidisciplinary Team

Variable	Extraction Group (n = 54)	Nonextraction Group (n = 26)	*P* Value
Cardiac arrest	9.3 (5)	3.8 (1)	.39
Cerebrovascular accident, No. (%)	3.7 (2)	15.4 (4)	.06
Length of stay, median (IQR), d	11 (10.8)	10.5 (11)	.75
Inpatient mortality, No. (%)	13.0 (7)	19.2 (5)	.47
90-d mortality, No. (%)	22.2 (12)	38.5 (10)	.13
30-d readmission, No. (%)	14.8 (8)	7.7 (2)	.37
30-d reinfections, No. (%)	0 (0)	3.8 (1)	.15
90-d reinfections, No. (%)	1.9 (1)	3.8 (1)	.61

Abbreviation: IQR, interquartile range.

## DISCUSSION

This single-center retrospective cohort study reports on 80 patients with suspected CIEDIs managed by a multidisciplinary endocarditis team. While multidisciplinary management of CIEDIs has been advocated by multiple professional organizations, few data have been published about this approach. Our study is unique in that it combines a multidisciplinary endocarditis team providing treatment for CIEDI with assessment of the impact on patient care processes and outcomes. There are several notable findings from this study. Over two-thirds (67.5%) of patients underwent device extraction, >5 times the rate reported in recent population studies [5, 6]. The median time to extraction after admission to our institution (3 days) and overall (5 days) was also very low. Forty-two point five percent of the cohort was transferred to our institution from another facility for management of suspected CIEDI, which likely increased the extraction rate compared with population-based studies, but the extraction rate and speed to extraction are still notable given the complex and critically ill nature of this cohort. Only 8 (10%) patients with definite CIED/IE did not undergo device removal, and no patients with definite pocket/generator infections had their device retained. Of these 8 patients, family meetings were held with 4, who ultimately elected to transition to comfort measures. Among the other 4 patients, 2 patients were noted to have echodensities on their CIED leads, but there was debate within the MDET as to whether this represented chronic thrombus or vegetation. In both patients, the risk of extraction was felt to be very high, and given the diagnostic uncertainty the recommendation was for medical management with close follow-up. One patient was offered device removal but declined, instead choosing medical therapy only. The last patient had concomitant aortic valve endocarditis with an aortic root abscess and was deemed not a candidate for valve surgery. Given the uncertain benefit of removing the CIED while not draining the aortic root abscess, CIED extraction was not pursued. In total, 35 patients met criteria for definite CIED/IE and 27 underwent device removal, for an extraction rate of 77.1%, again far above rates reported in the literature. Fewer patients with possible CIED/IE underwent device extraction, which was due to the uncertainty surrounding the diagnosis. Although causation cannot be proven in a retrospective cohort with this potential referral bias, the use of a multidisciplinary endocarditis team was associated with high rates of expedient extraction.

PET-CT was not routinely available in a timely fashion, and certain patient factors limited its use as the nuclear medicine suite at the authors’ institution is located in an ambulatory setting and patients must be clinically stable for transportation. However, the rates of TEE were also low, at 49% for the entire cohort and 46% for the nonextraction group. Historically, obtaining TEEs at the authors’ institution has been a logistical challenge. No procedure rooms or sedation-trained nursing staff are dedicated to performing TEEs, and this must be arranged ad hoc, typically by an on-call cardiology fellow when a TEE is requested. Consequently, once a diagnosis of lead or valve vegetation is made by TTE, TEE is often deferred. For patients whose devices were not extracted, all TTEs were reviewed by cardiologists on the MDET to determine whether the additional information that could be gained by performing a TEE outweighed the risk of the procedure. However, it is possible that the number of lead and valve vegetations in the nonextraction group was underestimated given that over half of these patients did not undergo TEEs.

Five (19.2%) patients whose devices were retained (including 3 with definite CIEDI) were transitioned to suppressive oral antibiotics after completion of their initial antibiotic course for a median duration of 349 days at the time of last follow-up. No patients on suppressive antibiotic therapy were readmitted for infection during the study period. There are few data to guide providers as they consider the role of suppressive antibiotics. The 2020 EHRA guidelines do suggest consideration of suppressive treatment in patients with definite CIED/IE and retained devices, but this recommendation is based on expert opinion [3]. Small retrospective case series have suggested that suppressive antibiotics can be well tolerated with relatively low rates of relapsed infection [8, 13]. Given the ambiguity in guidelines and the literature, decisions about the role of suppressive antibiotics are another facet of CIED care that can likely be enhanced by formal multidisciplinary endocarditis team management. Our strategy is to offer patients with definite CIED/IE whose devices are retained the option of suppressive oral antibiotic therapy, and then we use shared decision-making regarding whether to proceed with this approach.

It is notable that over half of the patients whose CIEDs were extracted did not require placement of a new device during the same hospitalization or follow-up, raising questions about the initial necessity of the device. It is also notable that well over half the devices replaced during the same hospitalization were replaced with leadless or subcutaneous devices that are associated with lower rates of endovascular infection than traditional transvenous devices [14, 15]. In total, 83.3% of patients who had a CIED extracted either did not have a new device placed or had a leadless pacemaker implanted upon discharge. These results highlight that the time of device extraction is an ideal opportunity to re-evaluate the original indications for the CIED and type of device to use next. The findings also raise the question of whether the involvement of a formal multidisciplinary endocarditis team in the management of CIEDIs may improve the decision-making process around ongoing need for CIEDs and find ways to mitigate the risk for subsequent infection when a CIED is needed. During our multidisciplinary conferences, the team always discusses whether patients with CIEDIs still have indications for device placement. If there is still an indication, the group will consider a leadless or subcutaneous device, if appropriate, and implement other measures to mitigate the risk of reinfection. For example, in patients with *Staphylococcus aureus* CIEDIs, we perform decolonization with chlorhexidine baths and nasal mupirocin treatments before reimplantation [16].

The in-hospital and 90-day mortality rates in both groups are higher than the ∼9% in-hospital and 10% early mortality previously reported [5]. This is likely in part due to our institution's role as a tertiary care referral center that accepts the most complex CIEDIs and the high degree of critical illness in the cohort. Additionally, due to delays in the transfer process, there was an increased median time to device removal in transferred individuals. Given the retrospective nature of the data, and some patients likely not undergoing extraction due to significant burden of comorbidities or low estimated life expectancy, it is difficult to draw strong conclusions about the improved mortality in the extraction group. However, it is worth noting that these results are consistent with other published literature that overall suggests lower mortality in CIEDIs when the device is extracted, and the difference in 1-year mortality between the extraction group (34%) and nonextraction group (70%) was large.

### Limitations

Our study is limited by its retrospective, single-center design. Additionally, patients were only included if they were discussed by the multidisciplinary endocarditis team, and it is possible that there were other suspected CIEDIs admitted to our institution that were excluded. Access to outside hospital records was limited, and patients presented to our institution from a wide catchment area. Therefore, it is possible that other post-discharge complications occurred and were not captured by the study investigators. Patients with suspected CIEDI in the setting of prosthetic valves or LVADs were excluded, and this may have also impacted the rates of extraction and clinical outcomes compared with other studies with different exclusion criteria. It is also possible that some of the cases of retained CIEDs were not true infections, and these patients may have had thrombi on their leads/valves or bacteremia without infection of their device. The relatively small numbers of patients also make it difficult to determine the significance of the higher rate of CVAs in the device retention group, and 3 of these patients had alternative explanations for their strokes. Finally, although the extraction and nonextraction cohorts were similar in many ways, due to the retrospective nature of the study it is possible that patients whose devices were retained were sicker at baseline, which may have contributed to the higher 1-year mortality in this group.

## CONCLUSIONS

CIEDI remains a serious complication with significant associated morbidity and mortality. Despite consensus management guidelines from several professional societal organizations, many patients do not undergo indicated device extractions. Given the complexity and acuity of this patient population, it will be difficult to achieve 100% compliance with societal guideline recommendations, especially given the nuances of some of the management scenarios. However, implementation of a multidisciplinary CIEDI team was associated with a 77.1% rate of device extraction for definite CIED/IE and a 100% rate of extraction for definite pocket/generator infections, far greater than the rates reported in recent studies. Additionally, the CIEDI team was associated with a lower median time to extraction: 3 days from admission to our institution and 5 days from presentation overall. In summary, the use of a multidisciplinary endocarditis team was associated with high rates of expedient device extraction, ensuring comprehensive care and reevaluation of the original indications for the CIED. This approach allows for tailored decisions regarding reimplantation, including consideration of leadless or subcutaneous devices where appropriate, and measures to mitigate reinfection risk, such as *S. aureus* decolonization protocols.

## Supplementary Material

ofaf148_Supplementary_Data

## Supplementary Data

Supplementary materials are available at *Open Forum Infectious Diseases* online. Consisting of data provided by the authors to benefit the reader, the posted materials are not copyedited and are the sole responsibility of the authors, so questions or comments should be addressed to the corresponding author.
