# Supplementary material for: Management of Cardiovascular Implantable Electronic Device Infection Utilizing a Multidisciplinary Team: A Retrospective Cohort Study
Source: Open Forum Infect Dis. 2025 Mar 10;12(4):ofaf148. doi: 10.1093/ofid/ofaf148 (PMC11949750; doi:10.1093/ofid/ofaf148)
Supplement: ofaf148_Supplementary_Data [file ofaf148_supplementary_data.zip › CIEDI Supplemental Table 1.docx]

Supplemental Table 1. Demographic and outcomes data for patients who underwent extraction for definite or possible CIED/IE and patients who underwent extraction for isolated pocket infections.

| **Variable** | **Definite or Possible CIED/IE**  **N = 34** | **Pocket Infections**  **N = 18** | **P Value** |
| --- | --- | --- | --- |
| Age, Median (IQR) | 65 (54.8 – 72.8) | 71 (56.3 – 79.5) | p = 0.28 |
| Pittsburgh Bacteremia Score, Median (IQR) | 0 (0 – 2) | 0 (0 – 0) | p = 0.01 |
| Bacteremia, % (n) | 85.3 (29) | 16.7 (3) | p < 0.0001 |
| Persistent Bacteremia, % (n) | 35.3 (12) | 0 (0) | p = 0.004 |
| Cerebrovascular Accident, % (n) | 5.9 (2) | 0 (0) | p = 0.30 |
| ICU Stay, % (n) | 38.2 (13) | 27.8 (5) | p = 0.46 |
| Length of ICU Stay, Median (IQR) | 10 (5.3 – 14.3) | 8 (6 -18) | p = 0.73 |
| Mechanical Ventilation, % (n) | 29.4 (10) | 16.7 (3) | p = 0.32 |
| Vasopressor Requirement, % (n) | 20.6 (7) | 5.6 (1) | p = 0.16 |
| Acute Renal Failure Requiring RRT, % (n) | 8.8 (3) | 5.6 (1) | p = 0.68 |
| Length of Stay, Median (IQR) | 14 (8.3 – 22.8) | 8 (6 – 10.3) | p = 0.03 |
| Inpatient Mortality, % (n) | 17.6 (6) | 11.1 (2) | p = 0.54 |
| 90-Day Mortality, % (n) | 20.6 (7) | 33.3 (6) | p = 0.32 |
