# Supplementary material for: Management of Cardiovascular Implantable Electronic Device Infection Utilizing a Multidisciplinary Team: A Retrospective Cohort Study
Source: Open Forum Infect Dis. 2025 Mar 10;12(4):ofaf148. doi: 10.1093/ofid/ofaf148 (PMC11949750; doi:10.1093/ofid/ofaf148)
Supplement: ofaf148_Supplementary_Data [file ofaf148_supplementary_data.zip › CIEDI Supplemental Table 2.docx]

Supplemental Table 2. Antimicrobial treatment data for patients with suspected cardiac implantable electronic device infections managed by a multidisciplinary team.

| **Variable** | **Extraction Group**  **N = 54** | **Non-Extraction Group**  **N = 26** | **P Value** |
| --- | --- | --- | --- |
| Days of Intravenous Antibiotic Therapy, Median (IQR) | 27 (33) | 17 (14.8) | 0.54 |
| Patients on Oral Antibiotics for Course Completion, % (n) | 37.0 (20) | 7.7 (2) | 0.006 |
| Total Days of Oral Antibiotic Course Completion Therapy, Median (IQR) | 9.3 (5) | 12 (4) | N/A |
| Patients on Oral Antibiotics for Suppressive Therapy, % (n) | 3.7 (2) | 19.2 (5) | 0.02 |
| Total Days of Oral Antibiotic Suppressive Therapy, Median (IQR) | 58 (66) | 349 (493.5) | N/A |
